# Synthesis and Physical Properties of Non-Crystalline Nylon 6 Containing Dimer Acid

**DOI:** 10.3390/polym11020386

**Published:** 2019-02-25

**Authors:** Ching-Nan Huang, Chang-Mou Wu, Hao-Wen Lo, Chiu-Chun Lai, Wei-Feng Teng, Lung-Chang Liu, Chien-Ming Chen

**Affiliations:** 1 Department of Textile Engineering, Chinese Culture University, Taipei 11114, Taiwan; chingnanhuang57@yahoo.com (C.-N.H.); deanwillmadeit2236@gmail.com (H.-W.L.); 2 Department of Materials Science and Engineering, National Taiwan University of Science and Technology, Taipei 10607, Taiwan; cmwu@mail.ntust.edu.tw; 3 Material and Chemical Research Laboratories, Industrial Technology Research Institute, Hsinchu 30011, Taiwan; wfteng@yahoo.com (W.-F.T.); markloukimo@yahoo.com.tw (L.-C.L.); cmchen101@yahoo.com (C.-M.C.)

**Keywords:** amphours nylon 6, transparency, ring-opening polymerization

## Abstract

In this study, a long carbon chain dimer acid is introduced into a nylon 6 structure and is copolymerized with different structural amines to produce amorphous nylon 6 by 4,4′-methylenebis(2-methylcyclohexylamine) (MMCA) in different copolymerization ratios. The effect of different structures and copolymerization ratios on the properties of nylon 6 is determined, along with the thermal properties, crystallinity, water absorption, dynamic mechanical properties, and optical properties. It is found that the melting point and the thermal cracking temperature Td10 of nylon 6 are respectively between 176 °C and 213 °C and 378 °C to 405 °C. The effect of introducing a bicyclohexane group containing a methyl side chain is greater than that of a meta-benzene ring, so COMM (synthesized by Caprolactam (C), dimer oleic acid (OA), and 4,4′-Methylenebis(2-methylcyclohexylamine) (MMCA)) has the lowest melting point, enthalpy, and crystallinity. As the copolymerization ratio increases, its thermal properties decrease. 10% is the lowest crystallinity. The amine structure containing a bicycloalkyl group has lower water absorption and a 10% copolymerization ratio gives the lowest water absorption. It contains the bicycloalkyl group, COM (synthesized by Caprolactam (C), dimer oleic acid (OA) and 4,4′-Methylenebis(cyclohexylamine) (MCA)), which has the highest loss modulus. The lowest loss modulus is noted for a copolymerization ratio of 7% and the value of tan δ increases as the copolymerization ratio increases. The introduction of nylon 6 with the bicycloalkyl groups, COMM and COM, significantly increases transparency. As the copolymerization ratio increases, the transparency increases and the haze decreases. The best optical properties are achieved for 10% copolymerization.

## 1. Introduction

Nylon 6 prepared by ring-opening polymerization of caprolactam is a common engineering polymer material. It has excellent properties, such as good chemical resistance, high mechanical properties, and high thermal stability. It is easily processed and is has been widely used in automotive, textile, packaging, biomedical applications, and solar cells. Although nylon 6 has excellent mechanical properties, good heat resistance, wear resistance and chemical solvent, and is very easily processed, the structure has a mercapto group so the hydrophilic group easily generates hydrogen bonds with water molecules, which means that nylon 6 readily absorbs water. Water absorption increases as the density of the amide group in the molecular structure increases. Dimensional changes occur when the material absorbs water, so the dimensional stability of the product is reduced. The tensile strength and bending strength decrease as the moisture absorption rate increases, which affects other properties of the product [1,2].

Generally, high molecular weight polymers contain crystalline and amorphous elements. The degree of crystallinity has a significant influence on the properties of high molecular weight polymers. Since the indoleamine groups in the nylon 6 molecular chain are arranged neatly, the intermolecular force is strong and the crystallinity is high. However, the high crystallinity of nylon 6 results in low transparency and high haze, limiting its applications in optical films, electrical devices and food packaging. The demand for high optical transparency polymers has increased in recent years. Therefore, the improvement of water absorption, transparency and haze for nylon 6 has become important for its applications in optical films and food packaging [3]. In this study, non-crystalline nylon 6 is prepared by copolymerization of dimer acid with different structural amines and different proportions of amines. Its thermal properties, optical transparency and hygroscopic characteristics are determined.

## 2. Materials and Methods

### 2.1. Materials

H: Hexanediamine (HMDA), C: 1,3-Cyclohexanebis(methylamine) (CHMA), M: 4,4′-Methylenebis(cyclohexylamine) (MCA), I:Isophorondiamine (IDPA), MM:4,4′-Methylenebis(2-methylcyclohexylamine) (MMCA), X: m-Xylylenediamine (XDA) were obtained from Acros (New Taipei City, Taiwan). These were purified by distillation. C: Caprolactam(CPL) and O: dimer oleic acid(OA)were obtained from commercial sources and purified by recrystallization.

### 2.2. Nylon Salts

The salt can be prepared by mixing the water or alcoholic solutions of the two components because of different hydrophobic property. Nylon salts were prepared by adding a 70% water solution of diamine to a 25% alcoholic solution of diacid at 60 °C and regulated the pH value to 7.5. The solutions of salt were clarified by active carbon powder at room temperature. The aqueous salt solutions were concentrated of about 50–60%.

### 2.3. Polymerization

**Figure 1:**
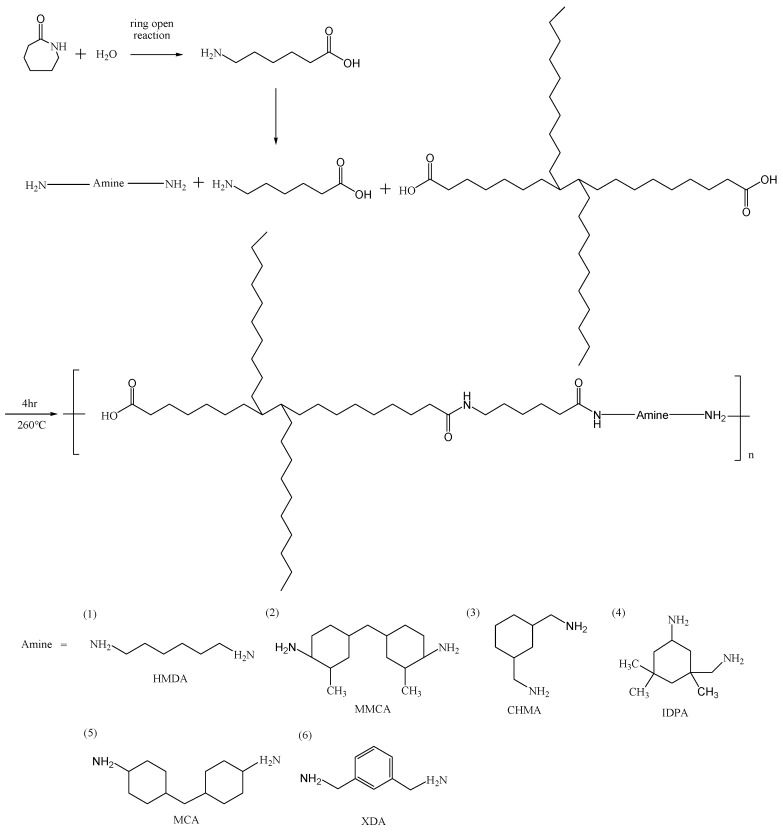
Synthesis of PA6 containing cyclic compounds.

The solution of salts proceeds polymerization of condensation. A 60% solution of each salt and Caprolactam(CPL) were heated for 3 h from temperature 30 to 220 °C in autoclave before prepolymerization of 2 h at 220 °C and atmospheric pressure at nitrogen atmosphere. The polymerization temperature was 260 °C and reaction 4 h until a specific viscosity was achieved shows in Figure 1. The different amines and acid should take different polymerization time 2–4 h [4]. The relative viscosity (RV) were measured with a Brookfield KV100 capillary viscometer at 25 °C.

### 2.4. Preparation of Films

The film samples were made by injection molding at temperatures of 230 to 260 °C above their melting points. The samples were maintained in a vacuum to prevent oxidation.

### 2.5. Thermal Analysis

Using Perkin-Elmer thermal analysis facilities proceed Differential Scanning Calorimerty (DSC), Thermogravimertic Analyzers (TGA) and Dynamic Mechanical Analysis (DMA) analysis. The samples were dehydrated to 800 ppm water content. DSC sample 10 mg of each film was placed on platinum sample holder at a heating rate of 10 °C/min under flowing nitrogen. The testing temperature was between room temperature to 250 °C. TGA sample 5 mg of each film was placed on platinum sample holder at a heating rate of 20 °C/min under flowing nitrogen. The testing temperature was between room temperature to 400 °C. DMA sample 10 mg of each film was placed on platinum sample holder at a heating rate of 10 °C/min under flowing nitrogen. The testing temperature was between −40 to 140 °C.

### 2.6. Sorption Isotherm

The samples that were used to determine the sorption properties were 10 mm square. These were immersed in deionized water at 28 °C for 24 h and 48 h. The weight was measured to calculate the absorption rate.

### 2.7. X-ray Diffraction

The X-ray diffraction patterns for the samples were obtained using a Bruker D8Advance diffractometer (Bruker, Hsinchu city, Taiwan) with an acceleration voltage of 40 kV and a current of 30 mA. Data was collected at room temperature in the 2θ range from 3° to 50°.

### 2.8. Optical properties

Based on ASTM D1003-61 (1997) method (Standard Test Method for Haze and Luminous Transmittance of Transparent Plastics), haze and transparency were obtained by haze meter (Labsanli WGT-S). The standard light were A (2856 K) and C (6447 K) at condition was 23 + 2 °C (73.4 + 3.6°F) and RH 50% + 5%. The thickness of sample was 15mm.

Haze=Td/Tt×100

*T*d: the transmittance of sample, Tt: the total transmittance.

## 3. Results and Discussion

### 3.1. Identification

**Figure 2:**
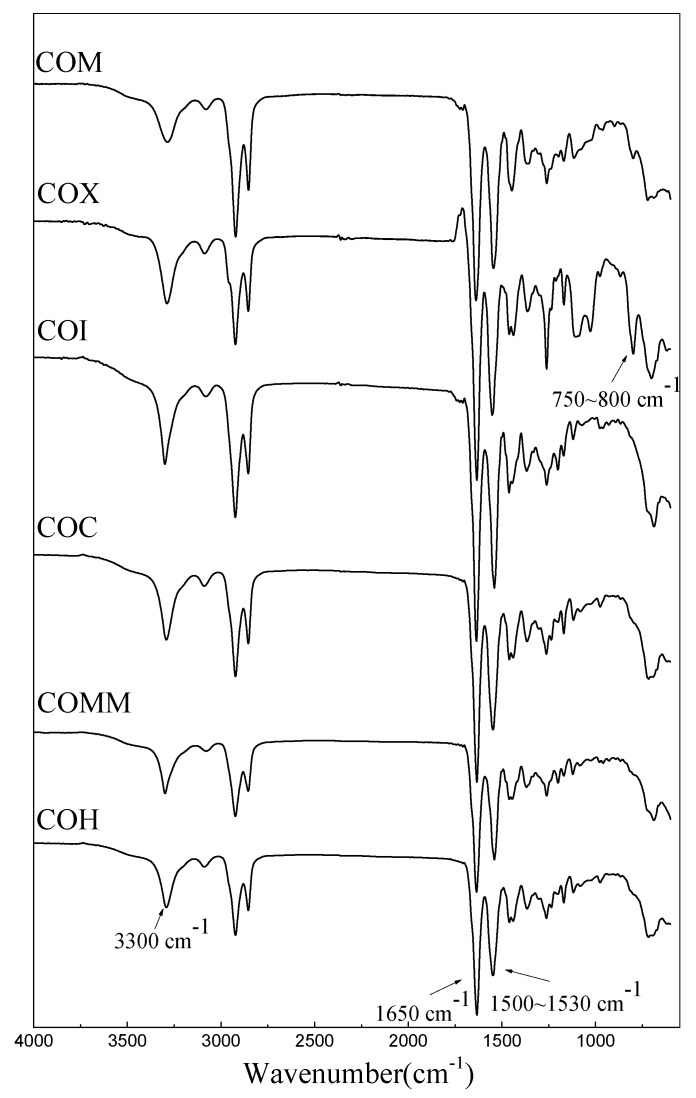
FTIR spectrum of PA6 containing cyclic compounds.

The functional group of the synthesized product was confirmed by Fourier Transform Infrared Spectroscopy (FT-IR) between 4000 and 550 cm^−1^, in order to determine the molecular structure of the synthesized product. Figure 2 shows the FTIR spectrum for nylon 6 with different copolymerization components. The nylon 6 peaks for different copolymerization components appear as the N–H stretching vibration peaks at 3300 cm^−1^, the C=O stretching vibration peak at 1650cm^−1^ and the C–N–H bending and the C–N stretching vibration peaks at approximately 1500~1530 cm^−1^. The COX shows a vibration peak for the benzene ring at 750–800 cm^−1^.

### 3.2. PA6-containing Cyclic Compounds

This study determines the physical effect of different types of amine PA6 copolymerization. The code for the composition, the composition of the RV and the amine group are shown in Table 1.

#### 3.2.1. Thermal Properties

**Figure 3:**
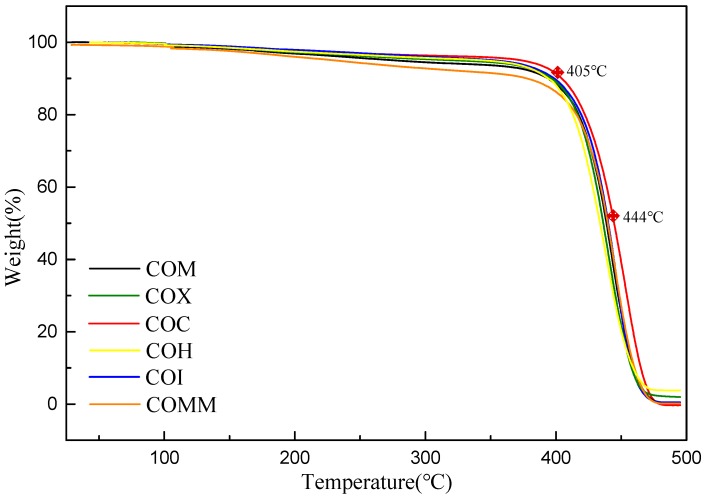
TGA Of PA6 containing cyclic compounds.

Figure 3 shows a TGA diagram for a COC nylon 6 copolymer under nitrogen gas. The respective values for *T*d10 and *T*d50 are 405 and 444 °C. The *T*d10 and *T*d50 values for each polymer are listed in Table 2. It is seen that *T*d10 is between 378 and 405 °C and *T*d50 is between 431 and 444 °C. COMM has the lowest value for *T*d10. Each polymer experiences thermal weight loss at more than 30 °C. It is also confirmed that each of the polymers has good heat resistance properties.

**Figure 4:**
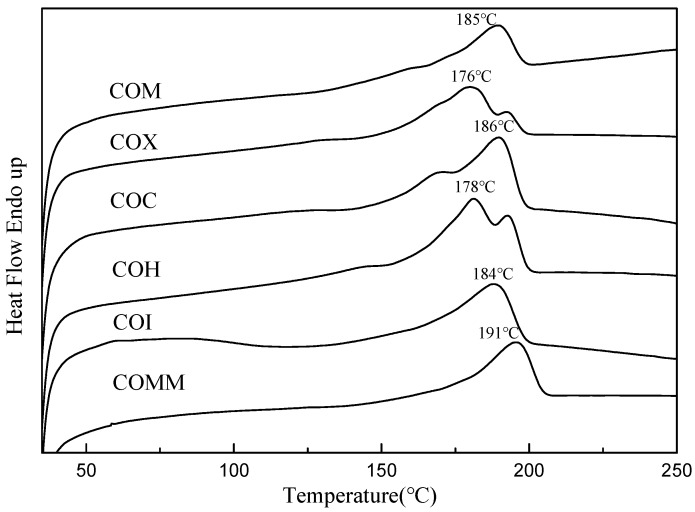
DSC for PA6 containing cyclic compounds.

The melting point, enthalpy and crystallinity of the synthetic products were measured using DSC, Figure 4 shows the DSC chart for different copolymerized nylon 6 samples. The *T*_m_ and crystallinity for each polymer are listed in Table 2. In terms of melting point, the main component of the polymer is nylon 6. The copolymerization of oleic acid dimer with 10% proportional long carbon chain and amines with different structures affects the melting point of nylon 6, so the melting point of the polymer is between 178 and 191 °C. In terms of crystallinity, the introduction of a bicyclohexane group containing a methyl side chain has a greater effect than a metacyclic benzene ring. COMM has the lowest crystallinity because its bulky structure disturbs the polymer molecular chain.

**Figure 5:**
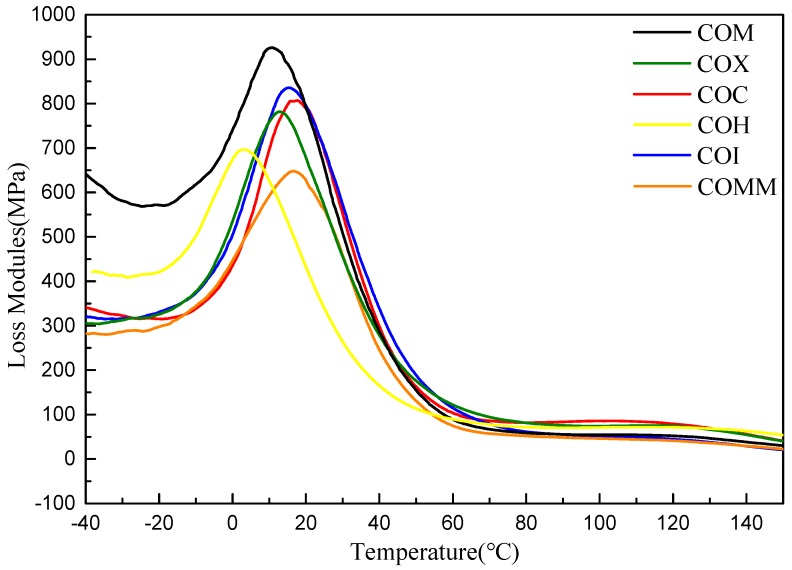
DMA for PA6 containing cyclic compounds.

**Figure 6:**
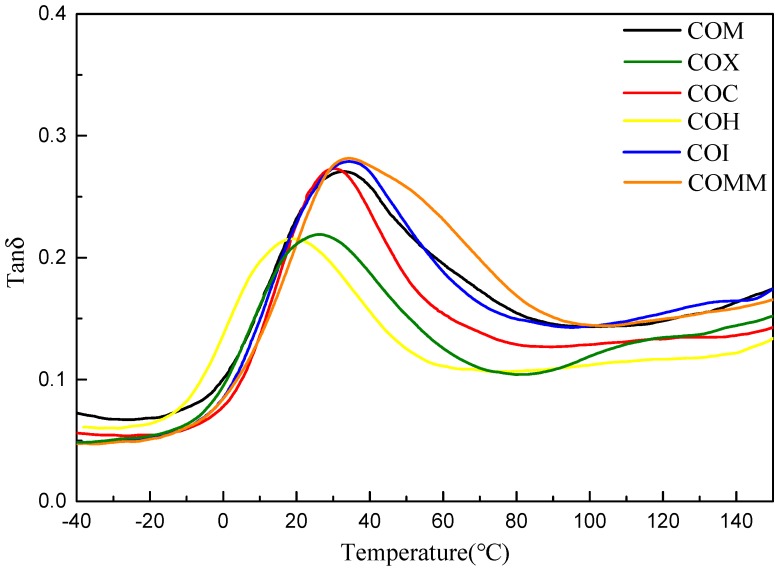
tanδ value for PA6 containing cyclic compounds.

This study uses a dynamic mechanical analyzer (DMA) to determine the storage modulus, the loss modulus and the tanδ for the synthesized product. The data shows the strength, viscosity, elasticity and glass transition temperature of the material as a function of temperature. Figure 5 shows the loss modulus diagram for nylon 6 with different copolymerization components. The figure shows that as the temperature rises close to its *T*_g_ point, the material becomes soft and the loss modulus gradually increases. The bicycloalkyl group containing COM has the highest loss modulus, mainly due to the absence of methyl side chains and meta benzene rings in the structure. The cycloalkane-containing methyl group and the meta-phenylene ring nylon 6 interfere with the movement of the molecular chain, resulting in a lower loss modulus. Figure 6 shows the tan δ value for nylon 6 with different copolymerization components. COMM and COI containing methyl side chains have a greater influence on nylon 6 and are very rigid, so the value of *T*_g_ is higher [5,6,7].

#### 3.2.2. Crystallinity

**Figure 7:**
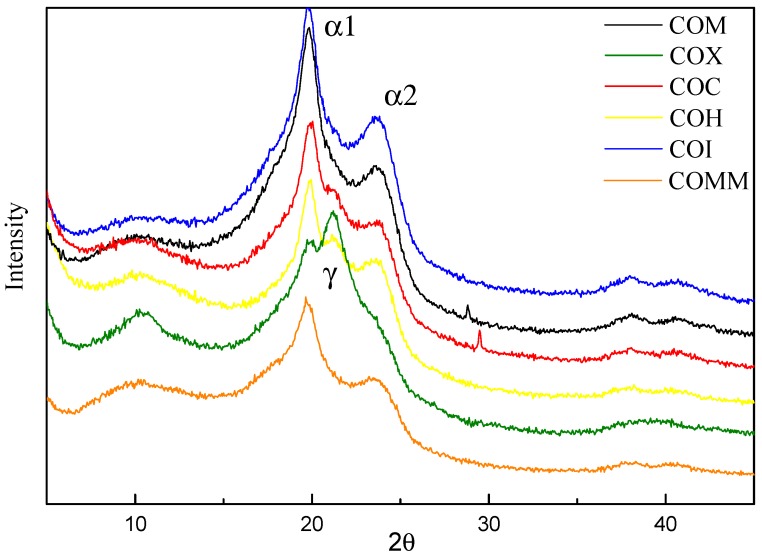
X-ray for PA6 containing cyclic compounds.

The crystal structure of the synthesized product was determined using an X-ray diffractometer at 2θ angles between 5° and 45°. Figure 7 shows the XRD spectrum for nylon 6 with different copolymerization components. The figure shows that the amine-containing cycloalkyl group-containing nylon 6 has an α-type doublet at 19.5° and 24°. COH also shares this feature. Because of the degree of freedom in the torsion for the cycloalkyl group, the crystal form retains a tighter alpha form. However, the COX containing a meta-benzene ring in the amine is crystallized at 21.3°~21.5° and the single peak of the γ form is dominant. There is loose γ crystallization due to the meta-benzene ring structure, which has less freedom of motion.

#### 3.2.3. Optical Properties

Table 3 shows the optical properties and water absorption for nylon 6 with different copolymerization components. The table shows that the nylon 6 copolymerization component can greatly improve the transparency due to the introduction of the bicycloalkyl groups, COMM and COM. It has better transparency and lower haze than monocycloalkane and the meta-benzene ring. The copolymerization of COH without any cycloalkyl and benzene ring structure results in low transparency and high haze. COMM produces the best transparency and lowest haze because there is minimal crystallization of nylon 6 due to the bulky structure of the dimethyl side chain and bicycloalkane in the structure. COM and COMM have lower water absorption because both contain a bicycloalkyl group and have more hydrophobic carbon chains, so they are more hydrophobic. COH has the highest water absorption because it is copolymerized without any cycloalkyl and benzene ring structure [8,9,10,11].

### 3.3. PA6-Containing Dimer Oleic Acid and 4,4′-Methylenebis(2-methylcyclohexylamine)

These experiments determine the effect of different copolymerization ratios for COMM on PA6. The code for the composition, the composition of the RV and the amine group are shown in Table 1 and Table 4.

#### 3.3.1. Thermal Properties

**Figure 8:**
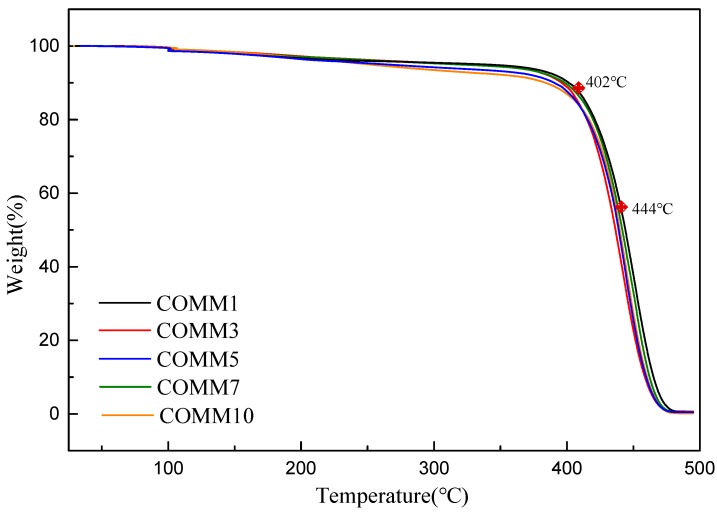
TGA of PA6 containing dimer oleic acid and 4,4′-Methylenebis(2-methylcyclohexylamine).

Figure 8 shows a TGA diagram of MMCA with different copolymerization ratios under nitrogen gas. COMM1 has the highest value for *T*d10 and *T*d50 are 402 and 444 °C. The *T*d10 and *T*d50 values for each polymer are listed in Table 5.

**Figure 9:**
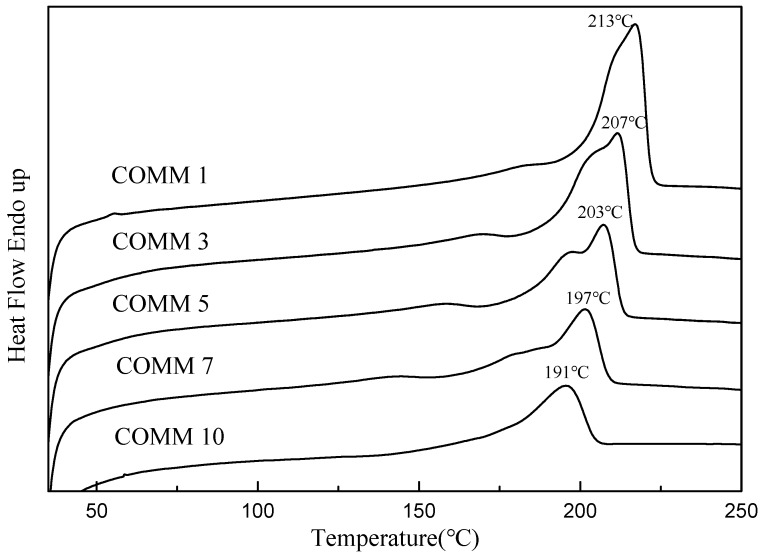
DSC of PA6 containing dimer oleic acid and 4,4′-Methylenebis(2-methylcyclohexylamine).

Figure 9 DSC diagram of MMCA with different copolymerization ratios. The figure shows that as the proportion of MMCA increases, the melting peak has a tendency to shift to the left, it is confirmed that the change of its crystal form, when the copolymerization ratio is 1%, it can be observed that the thermal properties of COMM are better than those of pure nylon, and have the best thermal properties. It is known from Table 5 that *T*d10 is at 378~402 °C, *T*d50 is at 436~444 °C, and all polymers have thermal weight loss above 350 °C. Therefore, each polymer has good thermal properties, and as the proportion of copolymerized components increases, the properties of melting point, enthalpy, crystallinity, etc. of COMM decrease, so the copolymerization ratio of 10% has a lower crystallinity [12,13,14,15].

**Figure 10:**
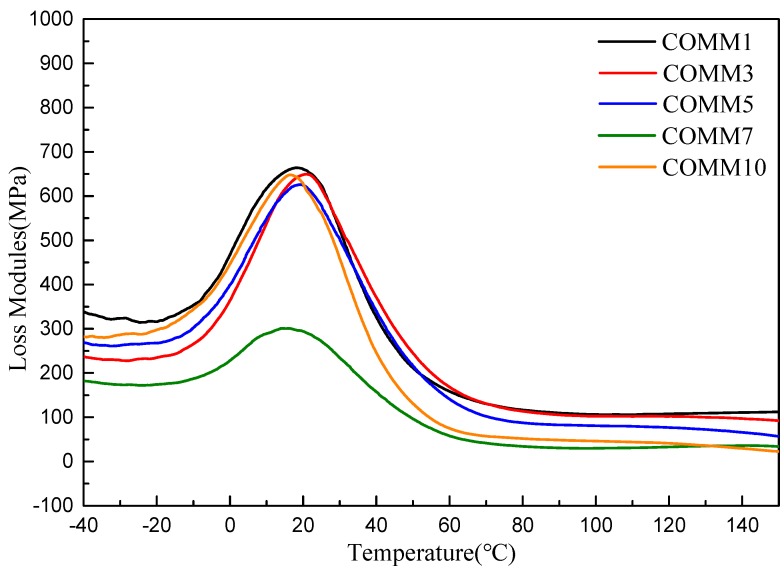
DMA of PA6 containing dimer oleic acid and 4,4′-Methylenebis(2-methylcyclohexylamine).

**Figure 11:**
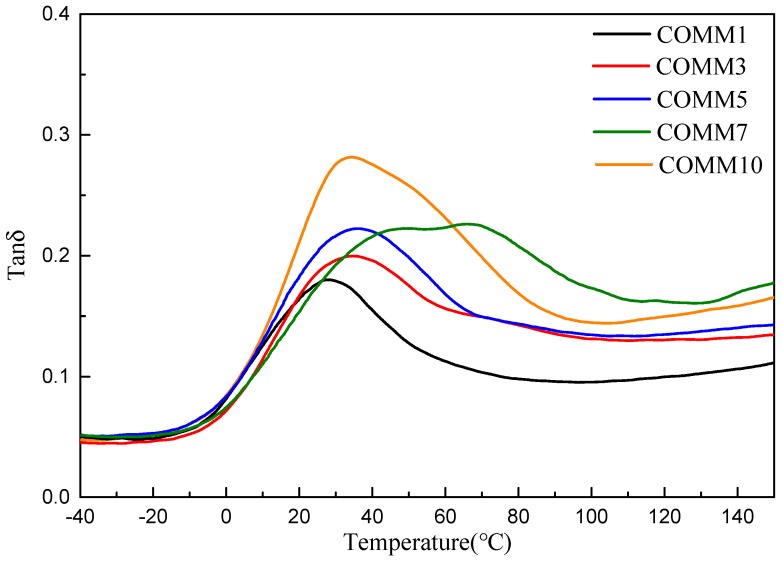
tanδ value for PA6 containing dimer oleic acid and 4,4′-Methylenebis(2-methylcyclohexylamine.

Figure 10 and Figure 11 show the loss modulus diagram and tanδ diagram of COMM with different copolymerization ratios. It is known from the figure that the copolymerization ratio of 7% has the lowest loss modulus, and at 10% of the copolymerization, the loss modulus increases due to low crystallinity. As the copolymerization ratio increases, the tanδ value increases, and the introduction of a long carbon chain gives nylon 6 a higher viscosity [16,17].

#### 3.3.2. Crystallinity

**Figure 12:**
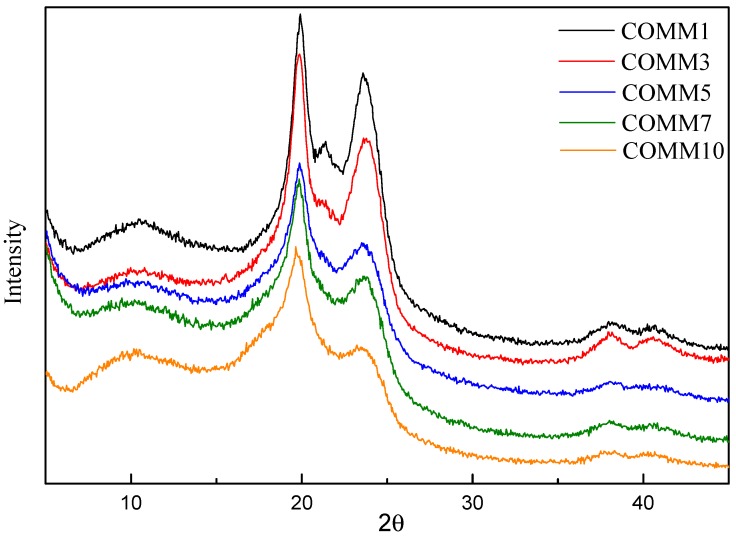
X-ray diffraction spectrum for PA6 containing dimer oleic acid and 4,4′-Methylenebis(2-methylcyclohexylamine).

Figure 12 shows the XRD spectrum for different copolymerization ratios of COMM. It is seen that 1%, 3%, 5%, 7%, 10% of COMM increases with the proportion of MMCA. Crystallization at 19.5° and 24.0° results in an α-type doublet. The α2 peak gradually disappears as the proportion of copolymerization increases. As the copolymerization ratio increases, the crystal structure is affected [18,19,20,21].

#### 3.3.3. Optical Properties

Table 6 shows the optical properties and water absorption for different copolymerization ratios for COMM. it is seen that as the copolymerization ratio increases, the transparency increases and the haze decreases, mainly due to the increase in the methyl side chain and the cycloalkyl group. COMM has the best optical properties when the copolymerized component is 10%. The water absorption decreases as the content of the bicycloalkyl group increases. The lowest water absorption occurs for a copolymerization ratio of 10% [22,23,24].

## 4. Conclusions

A ring-opening polymerization reaction for caprolactam is used and a dimer acid is copolymerized with different structural amines, to produce a series of non-crystalline nylon 6. Amine 6 containing a cycloalkyl group a crystal that is mainly in the form of α, which is also the case for COH. COX containing a meta-benzene ring in the amine is mainly in the γ form. Different copolymerized nylon 6 COMM has the lowest crystallinity. COM containing bicycloalkyl has a higher loss modulus than other nylon 6 containing methyl and benzene rings. COMM and COI containing a methyl group have a greater influence on nylon 6 and are very rigid, so the value for *T*_g_ is higher. The introduction of nylon 6 copolymerization component into COMM and COM containing bicycloalkyl groups can greatly improve the transparency, so it has better transparency and low haze than monocycloalkane and metacyclic benzene ring.

In the second part, different ratios of MMCA and dimerized oleic acid were prepared to synthesize different proportions of amorphous nylon 6 to investigate the effect of different ratios on the properties of nylon 6. Crystal structure at both 19.5° and 24.0° crystals showed an α-type doublet, and its peak gradually shifted from α-type to γ-type as the copolymerization ratio increased, confirming the influence on crystal structure. With regard to the thermal properties, the melting point, enthalpy, crystallinity, and other properties of COMM decrease with the increase of the proportion of copolymerization components. The optimal optical properties are obtained when the copolymerization component is 10%.

## Figures and Tables

**Table 1:** Composition of PA6 containing cyclic compounds.

Polymer	PA6 molar ratio	ACID molar ratio	BASE molar ratio	RV	NH2 meq/kg	*M*_w_	*M*_w_/*M*_n_
COM	CPL, 80%	OA, 10%	MCA, 10%	2.10	63	34556	1.42
COX	CPL, 80%	OA, 10%	XDA, 10%	2.31	63	32478	1.56
COC	CPL, 80%	OA, 10%	CHMA, 10%	2.05	83	34558	1.48
COH	CPL, 80%	OA, 10%	HMDA, 10%	2.44	66	38774	1.33
COI	CPL, 80%	OA, 10%	IDPA, 10%	2.25	60	32879	1.59
COMM	CPL, 80%	OA, 10%	MMCA, 10%	2.13	64	34226	1.49

C: Caprolactam(CPL), O: dimer oleic acid (OA), H: Hexanediamine (HMDA), C: 1,3-Cyclohexanebis(methylamine) (CHMA), M: 4,4′-Methylenebis(cyclohexylamine) (MCA), I:Isophorondiamine (IDPA), MM:4,4′-Methylenebis(2-methylcyclohexylamine) (MMCA), X: m-Xylylenediamine (XDA).

**Table 2:** Thermal properties of PA6 containing cyclic compounds.

Polymer	*T*_g_ °C	*T*_m_ °C	Δ*H* J/g	Crystallinity %	T_d_10 °C	T_d_50 °C
COM	32.8	185	24.0	10.0	395	438
COX	25.9	176	26.7	11.1	398	436
COC	30.1	186	23.4	9.7	405	444
COH	19.4	178	33.8	14.0	394	431
COI	34.7	184	23.8	9.9	397	440
COMM	34.2	191	21.6	9.0	378	439

*T*_g_: glass transition measured by DMA, Crystallinity: the value of 100% crystalline material’s melting heat is 240.

**Table 3:** Transparency and water absorption of PA6 containing cyclic compounds.

Polymer	Transparency (%)	Haze thickness*0.3 mm (%)	Water Absorption 24 h (%)	Water Absorption 48 h (%)
COM	87.5	32.0	1.07	1.31
COX	85.8.	39.4	1.51	2.13
COC	87.9	52.4	1.29	1.62
COH	81.9	75.0	1.57	2.21
COI	87.3	39.4	1.30	1.62
COMM	88.8	29.2	1.24	1.42

**Table 4:** Composition of PA6 containing dimer oleic acidand. 4,4′-Methylenebis (2-methylcyclohexylamine).

Polymer	PA6 molar ratio	ACID molar ratio	BASE molar ratio	RV	NH2 meq/kg	*M*_w_	*M*_w_/*M*_n_
COMM1	CPL, 98%	OA, 1%	MMCA, 1%	2.35	55	38621	1.3
COMM3	CPL, 94%	OA, 3%	MMCA, 3%	2.28	58	37552	1.45
COMM5	CPL, 90%	OA, 5%	MMCA, 5%	2.25	61	37421	1.47
COMM7	CPL, 86%	OA, 7%	MMCA, 7%	2.20	62	35223	1.47
COMM10	CPL, 80%	OA, 10%	MMCA, 10%	2.13	64	34226	1.49

**Table 5:** Thermal properties of PA6 containing dimer oleic acid and 4,4′-Methylenebis(2-methylcyclohexylamine).

Polymer	*T*_g_ °C	*T*_m_ °C	Δ*H* J/g	Crystallinity %	*T*d10 °C	*T*d50 °C
COMM1	27.7	213	97.2	40.5	402	444
COMM3	34.7	207	36.1	15.0	397	436
COMM5	36.6	203	33.5	13.9	387	438
COMM7	47.7	197	23.3	9.7	399	441
COMM10	34.2	191	21.6	9.0	378	439

*T*_g_: glass transition measured by DMA, Crystallinity: the value of 100% crystalline material’s melting heat is 240.

**Table 6:** Transparency and water absorption for PA6 containing Octadecadienoic acid and 4,4′-Methylenebis(2-methylcyclohexylamine).

Polymer	Transparency (%)	Haze thickness*0.3 mm (%)	Water Absorption 24 h (%)	Water Absorption 48 h (%)
COMM1	65.8	89.6	2.35	2.84
COMM3	76.9	84.6	1.78	2.61
COMM5	84.7	73.5	1.52	2.32
COMM7	87.1	41.9	1.22	1.84
COMM10	88.8	29.2	1.24	1.42
